# The utility of diffusion-weighted imaging in patients with spinal cord infarction: difference from the findings of neuromyelitis optica spectrum disorder

**DOI:** 10.1186/s12883-022-02903-y

**Published:** 2022-10-11

**Authors:** Makoto Kobayashi

**Affiliations:** Department of Neurology, Asahi General Hospital, 1326 I, Asahi, Chiba 289-2511 Japan

**Keywords:** Diffusion-weighted imaging, Magnetic resonance imaging, Neuromyelitis optica spectrum disorder, Spinal cord infarction

## Abstract

**Background:**

Magnetic resonance imaging (MRI) plays a crucial role in diagnosing spinal cord infarction (SCI). However, the findings are often indistinguishable from those of other intramedullary diseases, such as neuromyelitis optica spectrum disorder (NMOSD). Although diffusion-weighted imaging (DWI) is a promising technique, the utility for discriminating SCI from NMOSD remains unclear because the DWI findings of acute NMOSD lesions have not been investigated in detail.

**Methods:**

Clinical and MRI findings were retrospectively evaluated in 15 and 12 patients with acute SCI and NMOSD, respectively. First, clinical characteristics were compared between the SCI and NMOSD groups. Second, MRI abnormalities were examined to find differences between these groups. Third, in the SCI group, factors influencing T2 and DWI abnormalities were analyzed using the mixed-effects logistic regression analysis.

**Results:**

The proportion of female patients was higher in the NMOSD group (92%) than in the SCI (40%). The time from symptom onset to nadir was smaller in the SCI group (median [interquartile range]; 4 [0.1–8.3] hours) than in the NMOSD (252 [162–576]). On T2-weighted images, SCI lesions had smaller length than NMOSD (2 [1–2] and 5 [2–7] vertebral segments, respectively). Focal lesions within the T9–L2 level were found only in patients with SCI. DWI hyperintensity was observed both in the SCI (frequency, 100%) and NMOSD (60%) groups. On apparent diffusion coefficient (ADC) maps, the hyperintensities of SCI had corresponding hypointensities, whereas those of NMOSD were isointense and a large portion of NMOSD lesions had hyperintense signals. Owl’s eyes sign and pencil-like hyperintensity, typically reported as T2 findings suggestive of SCI, were also found on DWI. Posterior linear hyperintensity was frequently detected on DWI in patients with posterior spinal artery infarction. The presence of MRI abnormality revealing SCI was modeled with the time from symptom onset, imaging sequence and plane, and affected vascular territory.

**Conclusions:**

DWI and ADC maps help distinguish SCI from NMOSD. The time from symptom onset, imaging sequence, and imaging plane should be considered when MRI findings are interpreted in patients with suspected SCI.

## Background

Spinal cord infarction (SCI) is a rare myelopathy that often causes severe motor, sensory, and autonomic disabilities. In the initial assessments for patients with suspected SCI, the rapidity of symptom progression, neurological deficits that assist lesion localization, and magnetic resonance imaging (MRI) findings are important for the diagnosis [1, 2]. However, the intramedullary lesions detected by conventional MRI are frequently nonspecific and indistinguishable from those of inflammatory myelopathies. Especially, neuromyelitis optica spectrum disorder (NMOSD) may mimic SCI because both diseases sometimes have longitudinally extensive lesions that are expansile and centered around the gray matter [3–5].

On T2-weighted images, SCI often presents with “owl’s eyes sign” and “pencil-like hyperintensity” when the infarct exists in the anterior spinal artery (ASA) region. Owl’s eyes sign is a pair of abnormal hyperintensities around the anterior horns, which are observed on the axial plane and may be associated with the vulnerability of motor neurons to ischemic damage [6]. The sign frequently appears as an intramedullary and anteriorly-located linear hyperintensity on the sagittal plane, namely pencil-like hyperintensity. Ischemia of the posterior spinal artery (PSA) is more infrequent than that of the ASA and has T2 linear hyperintensities that are located dorsally in the spinal cord [7–9]. In patients with NMOSD, cord lesions typically take the form of longitudinally extensive transverse myelitis (LETM) spanning ≥ 3 vertebral segments, which are hyperintense on sagittal T2-weighted images [10, 11]. Moreover, NMOSD lesions are named “bright spotty lesions” when the T2 intensity is equal to or stronger than that of the surrounding cerebrospinal fluid (CSF) without flow void effects [5]. These T2 findings suggesting SCI or NMOSD surely help in the diagnosis; however, only a few studies have compared the frequency of these T2 findings between patients with SCI and NMOSD [3, 5].

Diffusion-weighted imaging (DWI) is considered a promising technique for diagnosing SCI. Studies have reported that acute SCI was revealed as high-intensity lesions with corresponding low-intensity regions on apparent diffusion coefficient (ADC) maps, indicating diffusion restriction of water molecules in the tissue [12]. The effectiveness of early detection has been reported in case reports or series [12–15] despite technical difficulties in obtaining magnetic resonance images of the spinal cord, such as smallness of the lesion volume and artifacts resulting from the adjacent bone and pulsations of the CSF and blood [16, 17]. However, it remains unclear whether spinal DWI and ADC maps truly have the effectiveness in the usual clinical settings because information on DWI findings of other intramedullary diseases is insufficient. The findings of inflammatory spinal cord disease in the acute phase may be important when clinicians determine treatments for patients with spinal cord lesions that are not clearly diagnosed before the results of essential tests are obtained.

To investigate the utility of conventional MRI and DWI in diagnosing ischemic spinal stroke, 27 consecutive patients (15 and 12 patients with acute SCI and NMOSD, respectively) were analyzed. First, clinical findings were compared between the SCI and NMOSD groups. Second, the frequency of abnormal MRI findings, including owl’s eyes sign, pencil-like hyperintensity, and LETM ≥ 3 vertebral segments, was examined. Third, in patients with SCI, factors associated with the presence of T2 and DWI abnormalities were evaluated.

## Methods

For this retrospective study, clinical and MRI findings of patients with acute SCI and NMOSD were analyzed. Inpatients hospitalized at the neurology department of a tertiary referral hospital from April 2010 to July 2021 were enrolled. The diagnosis of SCI was based on all of the following items, which were according to the proposed criteria [18]: (1) acute nontraumatic myelopathy that reaches a nadir within 12 h or a severe disability status with rapid progression of < 12 h; (2) intramedullary T2 hyperintense lesions that are not caused by spinal cord compression; (3) presence of at least one of the specific abnormalities (intramedullary DWI hyperintensities with corresponding low-ADC regions, associated vertebral body infarction, and arterial dissection/occlusion near the intramedullary lesions) or CSF analysis suggesting a noninflammatory cause; and (4) absence of other possible diseases that can cause cord lesions. NMOSD was diagnosed based on the international consensus diagnostic criteria [10].

MRI examinations were performed on a 1.5 T system (Avanto, Siemens Medical Systems, Erlangen, Germany) using a spinal array coil. Conventional MRI was usually undertaken with turbo spin-echo sequences and following parameters: T2 axial images, flip angle (FA) 170°, repetition time (TR) 3500–4300 ms, echo time (TE) 82–96 ms, slice thickness (SL) 3–4 mm, number of acquisition (AC) 1, matrix 320, field of view (FoV) 180 mm; T2 sagittal, FA 170°, TR 3200–3500 ms, TE 81–104 ms, SL 3–4 mm, AC 1, matrix 384–448, FoV 280–320 mm; T1 axial, FA 135–170°, TR 550–620 ms, TE 8.7–10 ms, SL 3–4 mm, AC 1, matrix 320, FoV 180 mm; T1 sagittal, FA 140–150°, TR 540–585 ms, TE 9.2–12 ms, SL 3–4 mm, AC 1, matrix 384–448, FoV 280–320 mm. In rare cases, T2 axial images were obtained using a gradient-echo sequence. A single-shot echo-planer DWI sequence was performed in 3-scan trace mode with 2 b-values and following parameters: axial images, FA 90°, TR 3125–4860 ms, TE 84–90 ms, SL 3–4 mm, AC 1, matrix 144 × 256–184 × 256, FoV 130 × 260–180 × 240 mm^2^, minimum b value 0 s/mm^2^, maximum b value 800–1500 s/mm^2^; sagittal, FA 90°, TR 2400–3000 ms, TE 79–85 ms, SL 3–4 mm, AC 1, matrix 256 × 144–256 × 192, FoV 300 × 150–320 × 240 mm^2^, minimum b-value 0 s/mm^2^, maximum b-value 800–1500 s/mm^2^. The imaging location, sequence, and plane were selected depending on clinical requirements.

First, clinical characteristics of the SCI and NMOSD groups were compared. The characteristics were age, sex, hypertension, diabetes mellitus, dyslipidemia, atrial fibrillation, time from symptom onset to nadir, motor paresis (i.e., paraparesis, monoparesis, hemiparesis, and brachial diparesis), sensory disturbance, white blood cell (WBC) count and total protein of the CSF, CSF/blood glucose ratio, and ability to walk independently 1 month after symptom onset. Age, time from symptom onset to nadir, WBC count and total protein of the CSF, and CSF/blood glucose ratio were evaluated as numerical variables and the other items were categorical.

Second, on T2-weighted images, craniocaudal lesion length in each patient was compared between the SCI and NMOSD groups. In the situation of a patient having > 1 lesions, the longer or longest lesion was used. The distribution of cord lesions was also examined and compared between the groups when a specific vertebral level was suspected to be differently affected. The frequency of the following findings was examined: owl’s eyes sign, pencil-like hyperintensity, posterior linear hyperintensity (dorsally-located linear hyperintensity in the spinal cord), LETM ≥ 3 vertebral segments, and bright spotty lesion.

Subsequently, the frequency of DWI and ADC abnormalities was evaluated in both groups. When DWI hyperintensities were observed for the first time in each patient, mean ADC values were measured in the middle position of the hyperintensities and divided by mean ADC values of the adjacent normal cord (ADC ratios for DWI hyperintense lesions). For the NMOSD group, mean ADC values of lesions that were not hyperintense on DWI were also obtained and divided by mean ADC values of the normal adjacent cord (ADC ratios for NMOSD lesions that were not hyperintense on DWI). Moreover, the frequency of owl’s eyes sign, pencil-like hyperintensity, and posterior linear hyperintensity on DWI was examined. Spinal cord atrophy and gadolinium enhancement were assessed on T1-weighted images.

Third, in patients with SCI, T2 and DWI signal abnormalities within 7 days from symptom onset were evaluated and analyzed with the mixed-effects logistic regression analysis. MRI signal abnormality (presence or absence; absence was set as the reference level) was modeled using the following possible predictor variables: age (measured by years), sex (male or female; reference level, male), time from symptom onset (measured by hours), imaging sequence (T2 or DWI; reference level, T2), imaging plane (axial or sagittal; reference level, sagittal), and affected vascular territory (ASA, PSA, and ASA + PSA; reference level, ASA). The mixed-effects logistic regression analysis was performed with the random intercept of subjects and inclusion of predictor variables was determined using the likelihood ratio test and previous knowledge in the literature.

During statistical assessments, values were compared between the SCI and NMOSD groups using the Student’s t-test or Mann–Whitney U test. Similarly, frequencies were examined with the Fisher’s exact test. *P* values of < 0.05 were used to denote significance. Statistical calculations were performed with software R 4.1.3 and its lmerTest package [19, 20].

## Results

### Clinical findings in the SCI and NMOSD groups

During the analysis period, 27 patients were evaluated (15 and 12 consecutive patients with acute SCI and NMOSD, respectively). All patients with NMOSD were positive for serum anti-aquaporin 4 antibody. Age was not statistically different between the SCI and NMOSD groups (group, mean ± standard deviation; SCI, 61.8 ± 15.3 years; NMOSD, 59.5 ± 13) (Table 1). The proportion of female patients was higher in the NMOSD group (92%) than in the SCI (40%), whereas the frequency of hypertension, diabetes mellitus, and dyslipidemia was higher in the SCI group than in the NMOSD. The time from symptom onset to nadir was smaller in the SCI group (median [interquartile range]; 4 [0.1–8.3] hours) than in the NMOSD (252 [162–576]). The frequency of motor paresis and sensory disturbance did not reveal significant differences. WBC count of the CSF was higher in the NMOSD group (median [interquartile range]; 4.5 [3–15]/µl; mononuclear cell, 98.5 [93–100]%; polymorphonuclear, 1.5 [0–7]%) than in the SCI (1 [0–1]/µl; mononuclear, 100 [100–100]%; polymorphonuclear, 0 [0–0]%). Total protein in the CSF and CSF/blood glucose ratio were not different between these groups. CSF oligoclonal bands were measured in 2 and 8 patients with SCI and NMOSD, respectively; however, none exhibited positivity. For patients with SCI, the cause was presumed to be protruding aortic atheroma in 1, atrial fibrillation in 3, fibrocartilaginous embolus in 4, and undetermined in 7, based on the previous literature [21, 22]. Intravenous treatments for SCI were as follows: heparin in 2, argatroban in 4, and methylpredonisolone in 2. No specific intravenous treatment was administered in 9. Oral medications were antiplatelet and anticoagulant drugs in 10 and 5 patients, respectively. All patients with NMOSD were treated with intravenous methylpredonisolone 1000 mg/day for 3–5 days followed by oral predonisolone. In both groups, approximately one half of the patients could walk without assistance 1 month after symptom onset.

**Table 1:** Clinical characteristics of patients with SCI and NMOSD

	SCI	NMOSD	*p* value
Number of subjects	15	12	
Age (years)	61.8 ± 15.3	59.5 ± 13	0.69
Female	40%	92%	**0.014**
Hypertension	60%	17%	**0.047**
Diabetes mellitus	33%	0%	**0.047**
Dyslipidemia	87%	17%	**< 0.001**
Atrial fibrillation	27%	0%	0.11
Time from symptom onset to nadir (hours)	4 [0.1–8.3]	252 [162–576]	**< 0.001**
Motor paresis	80%	67%	0.66
Paraparesis	40%	42%	1
Monoparesis	27%	17%	0.66
Hemiparesis	7%	8%	1
Brachial diparesis	7%	0%	1
Sensory disturbance	100%	100%	1
CSF WBC count (/µL; NR, 0–5)	1 [0–1] (9)	4.5 [3–15] (10)	**0.0071**
CSF total protein (mg/dL; NR, 15–50)	48 [33–53] (9)	37.5 [35.3–56.5] (10)	1
CSF/blood glucose ratio	0.56 ± 0.08 (9)	0.56 ± 0.08 (9)	0.96
Ability to walk without assistance 1 month after symptom onset	47%	50%	1

Values are presented as number, percentage, mean ± standard deviation, or median [interquartile range]. Items revealing significant differences between the SCI and NMOSD groups have *p* values in bold face. When all subjects are not assessed, the numbers of available subjects are indicated in the parentheses

*CSF* Cerebrospinal fluid, *NMOSD* Neuromyelitis optica spectrum disorder, *NR* Normal range, *SCI* Spinal cord infarction, *WBC* White blood cell

### Comparison of MRI findings between the SCI and NMOSD groups

**Fig. 1:**
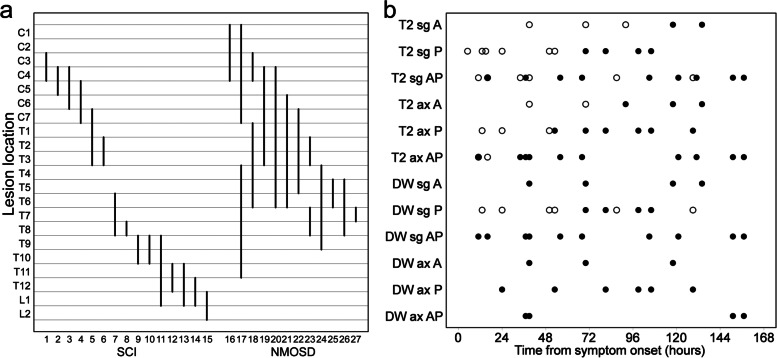
Lesion location and MRI positivity. **a** In patients with SCI and NMOSD, the craniocaudal distribution of lesions is presented based on the adjacent vertebral body segments. Focal lesions within the T9–L2 level were observed only in patients with SCI. **b** MRI findings indicating the positivity or negativity for SCI (closed or open circles, respectively) are classified according to the imaging sequence (T2 or DW), imaging plane (ax or sg), and affected vascular territory (A, P, or AP) and plotted as a function of time from symptom onset. Groups associated with T2 or sg have many open circles. A, anterior spinal artery; AP, anterior and posterior spinal arteries; ax, axial plane; DW, diffusion-weighted imaging; MRI, magnetic resonance imaging; NMOSD, neuromyelitis optica spectrum disorder; P, posterior spinal artery; SCI, spinal cord infarction; sg, sagittal plane

For patients with SCI, lesions were assessed on sagittal T1, T2, and DWI/ADC map images in 15 patients with axial T1 in 13, T2 in 15, and DWI/ADC map in 12. NMOSD lesions were imaged on sagittal T1 in 12, T2 in 12, and DWI/ADC map in 10. Moreover, they were assessed on axial T1 in 9, T2 in 12, and DWI/ADC map in 2. The craniocaudal locations of lesions in each patient are presented in Fig. 1a. For patients with SCI, lesions affected the ASA territory in 4, PSA in 5, and ASA + PSA in 6. None of the patients underwent spinal angiography and were proved to have arterial dissection or occlusion near the intramedullary lesions.

**Fig. 2:**
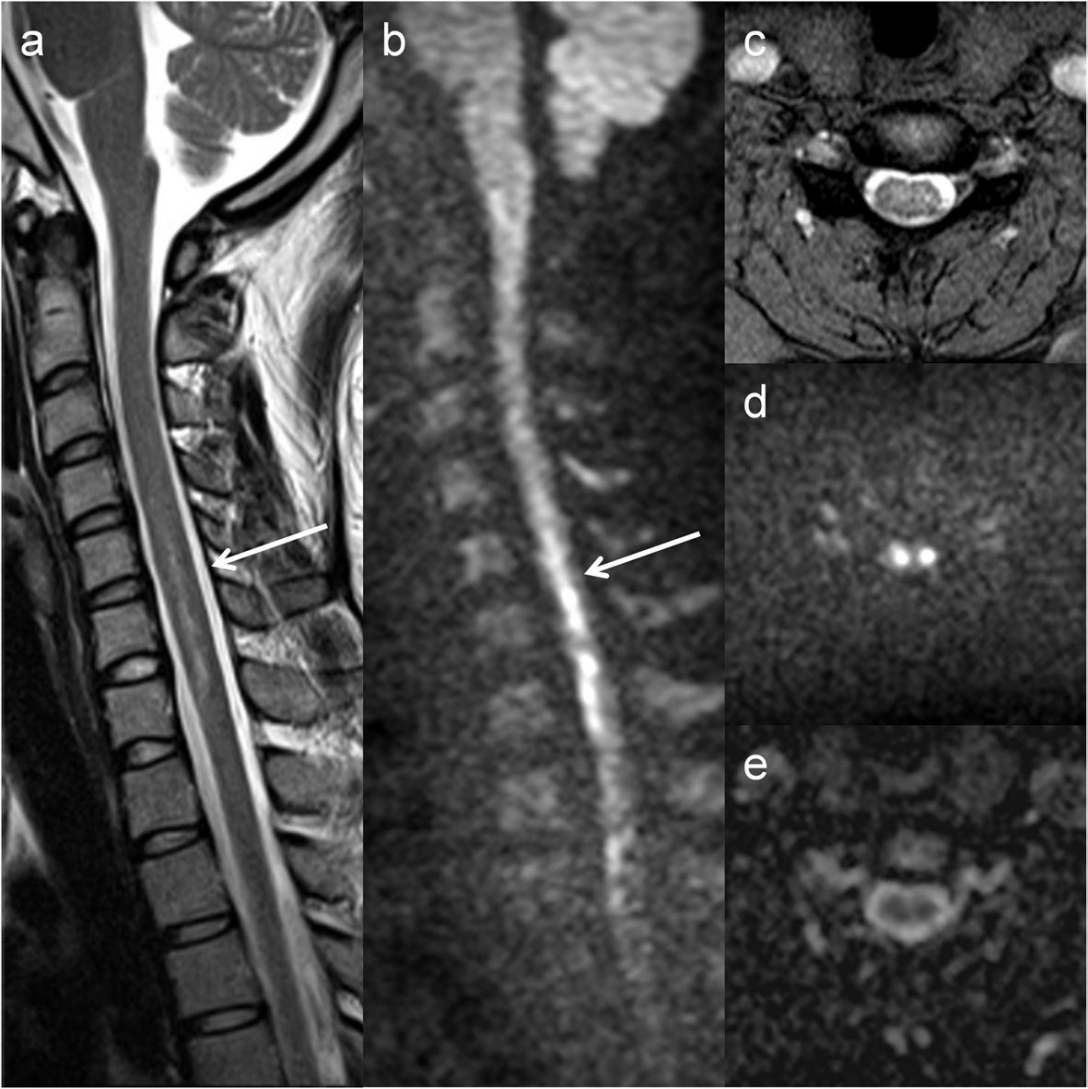
Pencil-like hyperintensity and owl’s eyes sign on DWI. A 21-year-old woman presented with muscle weakness in the upper limbs and back pain. MRI was performed 9 days after symptom onset and revealed a linear T2 abnormality spanning the C5–7 vertebral level (**a**). The linear abnormality, namely pencil-like hyperintensity, was also observed on DWI (**b**). At the C5/6 intervertebral level (arrows, a and b), mild T2 hyperintensities around the anterior horns were observed on the axial plane (**c**); moreover, the hyperintensities became a pair of apparent hyperintense lesions on DWI (**d**) and corresponded to owl’s eyes sign. The lesions on the axial DWI were hypointense on ADC maps (**e**). She was diagnosed with spinal cord infarction. ADC, apparent diffusion coefficient; DWI, diffusion-weighted imaging; MRI, magnetic resonance imaging

On T2-weighted images, lesion length was smaller in the SCI group (median [interquartile range], 2 [1–2] vertebral segments) than in the NMOSD (5 [2–7]) (Table 2). Focal involvement within the T9–L2 vertebral level was observed only in the SCI group (Fig. 1a) and its frequency of SCI was statistically higher than that of NMOSD. Owl’s eyes sign and pencil-like hyperintensity were observed in 2 and 1 patients with SCI, respectively (Fig. 2). Posterior linear hyperintensity was found selectively in 4 of 5 patients with PSA ischemia, whereas LETM ≥ 3 vertebral segments and bright spotty lesion were found only in the NMOSD group. Vertebral body infarction was observed in 1 patient with SCI.

**Table 2:** Comparison of spine MRI findings between patients with SCI and NMOSD

	SCI	NMOSD	*p* value
T2 hyperintense lesion	100%	100%	1
Lesion length (vertebral segments)	2 [1–2]	5 [2–7]	**0.0019**
Focal lesion within the T9–L2 level	47%	0%	**0.0081**
Owl’s eyes sign	13%	0%	0.49
Pencil-like hyperintensity	7%	0%	1
Posterior linear hyperintensity	27%	0%	0.11
LETM ≥ 3 vertebral segments	0%	67%	**< 0.001**
Bright spotty lesion	0%	67%	**< 0.001**
DWI hyperintense lesion	100%	60% (10)	**0.017**
DWI hyperintensity with low-ADC regions	100%	0% (10)	**< 0.001**
Owl’s eyes sign	25% (12)	0% (2)	1
Pencil-like hyperintensity	7%	0% (10)	1
Posterior linear hyperintensity	27%	0% (10)	0.11

Values are presented as percentage or median [interquartile range]. Items revealing significant differences between the SCI and NMOSD groups have *p* values in bold face. When all subjects are not assessed, the numbers of available subjects are indicated in the parentheses

*DWI* Diffusion-weighted imaging, *LETM* Longitudinally extensive transverse myelitis, *MRI* Magnetic resonance imaging, *NMOSD* Neuromyelitis optica spectrum disorder, *SCI* Spinal cord infarction

**Fig. 3:**
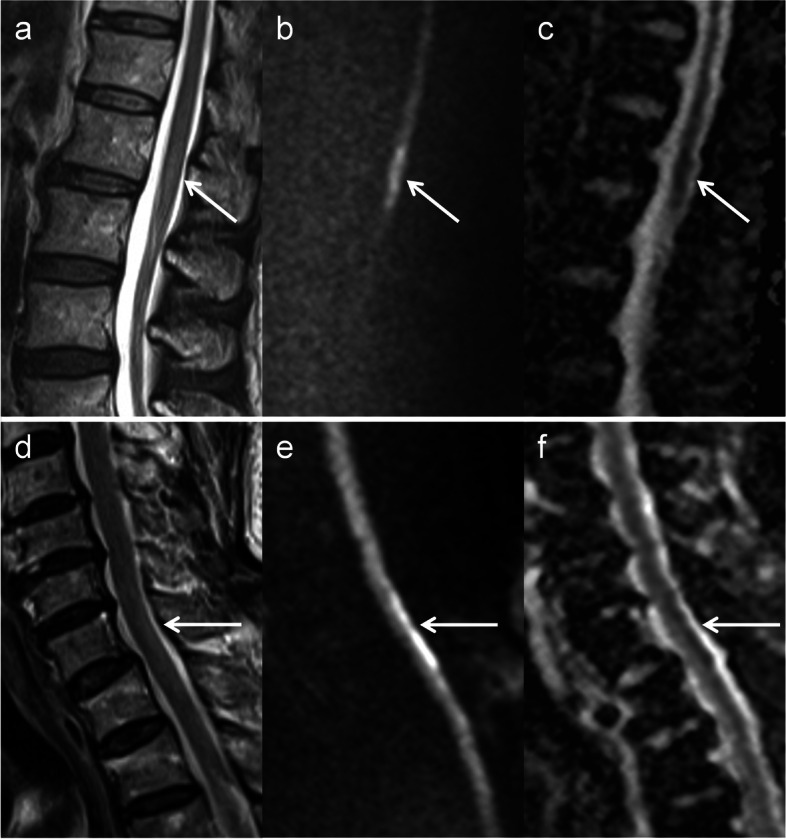
Early ischemic changes and posterior linear hyperintensity. MRI findings of a 73-year-old woman who noticed muscle weakness and paresthesia in the lower limbs were obtained 11 h from symptom onset (**a**–**c**). On T2-weighted images, the lesion was marginally high but not definitive (arrow, **a**); however, it was evident on DWI and ADC maps (arrows, **b** and **c**) and indicated spinal cord infarction. A 77-year-old man suddenly experienced back pain and gait difficulty and underwent MRI 4 days after symptom onset. Slight T2 hyperintensity was observed at the C7–T1 level of the cord (arrow, **d**), which was clearly high on DWI (arrow, **e**; posterior linear hyperintensity) and low on ADC maps (arrow, **f**). The lesion suggested ischemia of the posterior spinal artery. ADC, apparent diffusion coefficient; DWI, diffusion-weighted imaging; MRI, magnetic resonance imaging

**Fig. 4:**
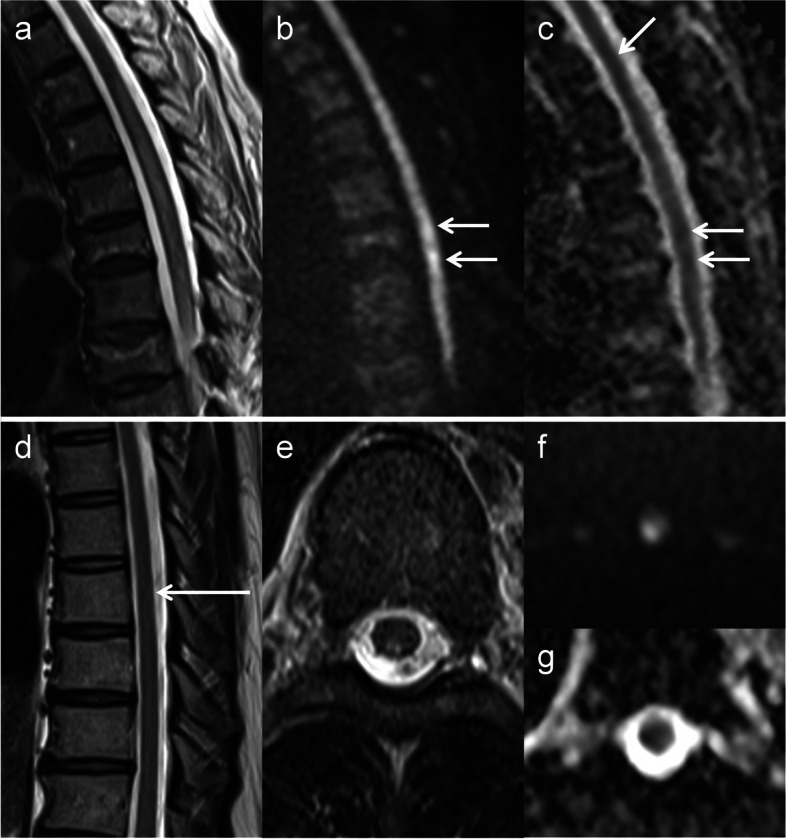
Representative DWI findings of NMOSD and small SCI. A 74-year-old woman noticed muscle weakness and numbness in the lower extremities. MRI was performed 5 days after symptom onset and revealed a T2 hyperintense lesion at the T4–9 level (**a**); moreover, small hyperintensities were observed around the T7 level on DWI (arrows, **b**). On ADC maps, the DWI hyperintensities were isointense (horizontal arrows, **c**) as compared with the intensity of the adjacent normal cord (oblique arrow, **c**) and surrounded by hyperintense regions. Her serum was positive for anti-aquaporin 4 antibody and these lesions were considered caused by NMOSD. Another patient is a 52-year-old man who presented with gait difficulty and mild muscle weakness in the right lower limb. Although MRI performed 5 days after symptom onset revealed no responsible lesion on the T2 sagittal image (**d**), an axial slice performed at the same occasion (indicated by an arrow in panel d; T8 level) demonstrated a right-sided lesion in the territory of the posterior spinal artery on T2-weighted imaging (**e**) and DWI (**f**). The lesion was hypointense on the ADC map (**g**) and he was diagnosed with spinal cord infarction. ADC, apparent diffusion coefficient; DWI, diffusion-weighted imaging; MRI, magnetic resonance imaging; NMOSD, neuromyelitis optica spectrum disorder

DWI hyperintensities were observed in all patients with SCI and in 6 out of 10 patients with NMOSD (Table 2). All DWI hyperintensities of SCI had corresponding low-ADC regions (Figs. 2 and 3); in contrast, those of NMOSD were isointense on ADC maps (Fig. 4). In 5 patients with NMOSD, the hyperintensities on DWI were considerably small as compared with those on T2-weighted images; moreover, the corresponding isointensities were surrounded by hyperintense regions on ADC maps (Fig. 4). NMOSD lesions were largely hyperintense on ADC maps in 9 patients. The ADC ratios for SCI and NMOSD lesions that were hyperintense on DWI were 0.71 [0.52–0.84] (median [range]) and 1.03 [0.98–1.06], respectively. The ADC ratios of NMOSD lesions that were not hyperintense on DWI were 1.3 [1.18–1.62]. Owl’s eyes sign and pencil-like hyperintensity were found as hyperintensity abnormalities on DWI with corresponding low-ADC regions in 1 and 2 patients with SCI, respectively (Fig. 2). Posterior linear DWI hyperintensity with low-ADC regions was observed in 4 of 5 patients with PSA ischemia (Fig. 3).

Spinal cord atrophy on T1 weighted-images was found in 1 patient with NMOSD. Gadolinium enhancement on T1 was observed in 1 of 3 patients with SCI and 5 of 10 patients with NMOSD.

### Factors associated with MRI abnormality in patients with SCI

T2 and DWI images obtained within 7 days from symptom onset were collected, totaling 96 series in 15 patients. As shown in Table 3, the presence of MRI abnormality was modeled with the time from symptom onset (odds ratio [95% confidence interval], 1.1 [1, 1.2]; estimate, 1 × 10^−1^; *p* value, 0.01), imaging sequence (3.6 × 10 [2.7, 4.8 × 10^2^], 3.6, 0.0066), imaging plane (2 × 10 [2, 2.1 × 10^2^], 3, 0.011), and affected vascular territory (PSA, 6.8 × 10^−1^ [1.4 × 10^−2^, 3.4 × 10], − 3.8 × 10^−1^, 0.85; ASA + PSA, 3.5 × 10^2^ [1.1, 1.2 × 10^5^], 5.9, 0.048). Age and sex did not have significant effects. For clarity, all data used for this analysis are presented as a function of time after classification according to the imaging sequence, imaging plane, and affected vascular territory (Fig. 1b). In a patient with unilateral PSA infarct, which was a focal lesion at the T8 level, sagittal T2 and DWI images performed 13, 87, and 129 h after symptom onset failed to detect the infarct; however, it was evident on the axial T2 and DWI performed 129 h after symptom onset (Fig. 4).

**Table 3:** Mixed-effects logistic regression analysis for MRI abnormality in patients with SCI

Predictor variable	Odds ratio [95% CI]	Estimate	SE	*z*	*p*
Intercept	**9.6 × 10**^−**5**^** [5.1 × 10**^−**8**^**, 1.8 × 10**^−**1**^**]**	− 9.2	3.8	− 2.4	0.016
Time from symptom onset	**1.1 [1, 1.2]**	1 × 10^−1^	4 × 10^−2^	2.6	0.01
Sequence (DWI)	**3.6 × 10 [2.7, 4.8 × 10**^**2**^**]**	3.6	1.3	2.7	0.0066
Imaging plane (Axial)	**2 × 10 [2, 2.1 × 10**^**2**^**]**	3	1.2	2.5	0.011
Vascular territory (PSA)	6.8 × 10^−1^ [1.4 × 10^−2^, 3.4 × 10]	− 3.8 × 10^−1^	2	− 1.9 × 10^−1^	0.85
(ASA + PSA)	**3.5 × 10**^**2**^** [1.1, 1.2 × 10**^**5**^**]**	5.9	3	2	0.048

Odds ratios of significant predictor variables are shown in bold face

*ASA* Anterior spinal artery, *CI* Confidence interval, *DWI* Diffusion-weighted imaging, *MRI* Magnetic resonance imaging, *PSA* Posterior spinal artery, *SCI* Spinal cord infarction, *SE* standard error

## Discussion

In this study, diffusion restriction was observed only in patients with SCI, although DWI hyperintensity was found in the lesions of both SCI and NMOSD. Moreover, owl’s eyes sign and pencil-like hyperintensity, which are typically reported on T2-weighted images, were also evident on DWI. Posterior linear hyperintensity was often found on T2 and DWI in patients with PSA ischemia. The factors associated with a higher possibility of detecting ischemic lesions were the longer time from symptom onset, DWI sequence (relative to T2), and axial plane (relative to sagittal). On T2-weighted images, focal lesions within the T9–L2 level were observed only in patients with SCI.

Diffusion restriction was observed in all patients with SCI; in contrast, no NMOSD lesions showed it. On ADC maps, in patients with NMOSD, the DWI hyperintensities were isointense and often surrounded by hyperintense regions. Although diffusion restriction has been reported as a useful finding for diagnosing SCI, information on other intramedullary spinal cord diseases is scarce. As for NMOSD, acute spinal cord lesions were described to have diffusion restriction in a review article [23]; moreover, the diffusivity of the chronic phase was increased [24, 25] and cerebral NMOSD lesions have increased diffusivity in the acute phase [26, 27]. However, to our knowledge, spinal DWI findings of the acute NMOSD lesions have not been analyzed as a cohort.

In this study, NMOSD lesions were largely hyperintese on ADC maps; in addition, only a small part of the lesions, which were hyperintense on DWI, had normal ADC values. Although the reason for the difference is unclear, it may be because not a single mechanism of tissue injury is active in NMOSD lesions. In a study, the lesions can be classified into six types, which are characterized by pathological findings, such as axonal degeneration, clasmatodendrosis, complement deposition, cystic formation, demyelination, granulocyte infiltration, and immunohistochemical reactivity for aquaporins 1 and 4 [28]. Considering the diverse and complex pathology, heterogeneity of the diffusivity can be observed in NMOSD lesions and a part of the lesion may show restricted diffusion. However, according to the results of this study, DWI findings of acute NMOSD lesions basically reveal normal or increased diffusivity. In clinical practice, although diffusion restriction in the spinal cord does not inevitably indicate SCI, its presence principally suggests SCI rather than NMOSD.

In patients with ASA ischemia, owl’s eyes sign and pencil-like hyperintensity were observed on DWI. Moreover, PSA ischemia often presented with a characteristic linear hyperintensity on sagittal DWI that was located posteriorly in the spinal cord. These DWI hyperintensities were low on ADC maps. Owl’s eyes sign and pencil-like hyperintensity have been reported as frequent T2 findings in patients with SCI [6]; however, owl’s eyes sign is not specific for SCI and can be observed in patients with a number of diseases, including amyotrophic lateral sclerosis [29], compressive myelopathy [30], myelitis due to enteroviruses 71 and D68 [31, 32] and West Nile virus [33], NMOSD [3, 4], poliomyelitis [34], and Werdnig–Hoffmann disease [35]. Pencil-like hyperintensity may be observed in similar diseases because lesions showing owl’s eyes sign are frequently expressed as pencil-like hyperintensity on the sagittal plane. However, the specificity of these findings on DWI remains unknown. In the cerebrum, DWI hyperintensity accompanied by the corresponding low-ADC region is more sensitive and specific for ischemia than T2 hyperintensity [36]; thus, the findings may be more specific for ischemia when they are observed on DWI than on T2. Posterior linear hyperintensity was often observed in patients with PSA ischemia, whereas it was not found in the NMOSD group. Although similar linear lesions on T2 may be observed in acquired immunodeficiency syndrome-related vacuolar myelopathy [37], copper deficiency [38], neurosyphilis [39], nitrous oxide intoxication [40], Sjögren syndrome [41], subacute combined degeneration of the spinal cord [42], and subacute myelo-optico-neuropathy (i.e., side effects of clioquinol) [43], posterior linear DWI hyperintensity that has low-ADC values is possibly more specific for SCI due to the same aforementioned reasons. Owl’s eyes sign, pencil-like hyperintensity, and posterior linear hyperintensity can be observed on DWI with the corresponding low-ADC region and may contribute to the diagnosis of SCI.

In patients with SCI in the acute phase (within 7 days from symptom onset), DWI more likely detected ischemic lesions than T2-weighted imaging. Furthermore, the axial plane and longer time from symptom onset raised the possibility of detecting lesions. DWI was described to be more useful for detecting early ischemic changes in the spinal cord than T2-weighted imaging [16], and in this study the fact was statistically confirmed with other factors simultaneously analyzed. Regarding the difference in imaging planes, in the literature, SCI lesions not evident on the sagittal plane occasionally became apparent on the axial plane [16]. The predominance of the axial plane was statistically proven in this study, which was probably caused by partial volume effects when lesions were not fully captured in the sagittal plane. Therefore, axial imaging may be recommended even if MRI fails to detect lesions on the sagittal plane. As the longer time from symptom onset is a significant factor, follow-up MRI must be performed when initial MRI detects no abnormality. Lesions in the ASA + PSA territory were more likely detected than those in the ASA territory, indicating lager lesions can be more easily found than smaller ones. The time from symptom onset, imaging sequence, and imaging plane should always be considered when T2 and DWI findings are interpreted in patients with suspected SCI.

Focal lesions within the lower thoracic and lumbar level (T9–L2) were observed only in patients with SCI; moreover, lesion length in each patient was longer in patients with NMOSD than in those with SCI. In previous studies, NMOSD lesions were located mainly in the cervical and upper thoracic cord, whereas SCI often affected the lower thoracic and lumber spinal cord [3–5, 44]. These findings agree with our results, where SCI lesions were frequently observed focally within the T9–L2 level. Moreover, the longer length of NMOSD lesions is also compatible with the results in a previous study [5]. In addition to the findings of LETM ≥ 3 vertebral segments and bright spotty lesion, which have been considered useful findings for diagnosing NMOSD, information on the focal lesion within the lower thoracic and lumbar level will help distinguish SCI lesions from NMOSD.

This study has some limitations. Although most findings reported in this study were statistically examined, the number of patients was small to obtain definitive conclusions. Another limitation was that no patients with SCI underwent spinal angiography, which was probably associated with the absence of arterial abnormalities near the infarcted cord. The SCI diagnostic criteria of this study included time from symptom onset to nadir and DWI findings; hence, the statistical analyses regarding these items might have been affected by selection bias.

## Conclusions

In this study, we have demonstrated the utility of DWI and ADC maps. The presence of diffusion restriction indicated SCI rather than NMOSD. Owl’s eyes sign, pencil-like hyperintensity, and posterior linear hyperintensity were observed on DWI with corresponding low-ADC regions. These DWI findings may help diagnose SCI. To enhance the utility of MRI for diagnosing SCI, the time from symptom onset, imaging sequence, and imaging plane should be considered when T2 and DWI findings are interpreted.

## Data Availability

The datasets used during the current study are available from the corresponding author on reasonable request.

## Abbreviations

AC: Number of acquisition
ADC: Apparent diffusion coefficient
ASA: Anterior spinal artery
CSF: Cerebrospinal fluid
DWI: Diffusion-weighted imaging
FA: Flip angle
FoV: Field of view
LETM: Longitudinally extensive transverse myelitis
MRI: Magnetic resonance imaging
NMOSD: Neuromyelitis optica spectrum disorder
PSA: Posterior spinal artery
SCI: Spinal cord infarction
SL: Slice thickness
TE: Echo time
TR: Repetition time
WBC: White blood cell
